# Supplementary figures and images for: BK channel density is regulated by endoplasmic reticulum associated degradation and influenced by the SKN-1A/NRF1 transcription factor
Source: PLoS Genet. 2020 Jun 5;16(6):e1008829. doi: 10.1371/journal.pgen.1008829 (PMC7299407; doi:10.1371/journal.pgen.1008829)

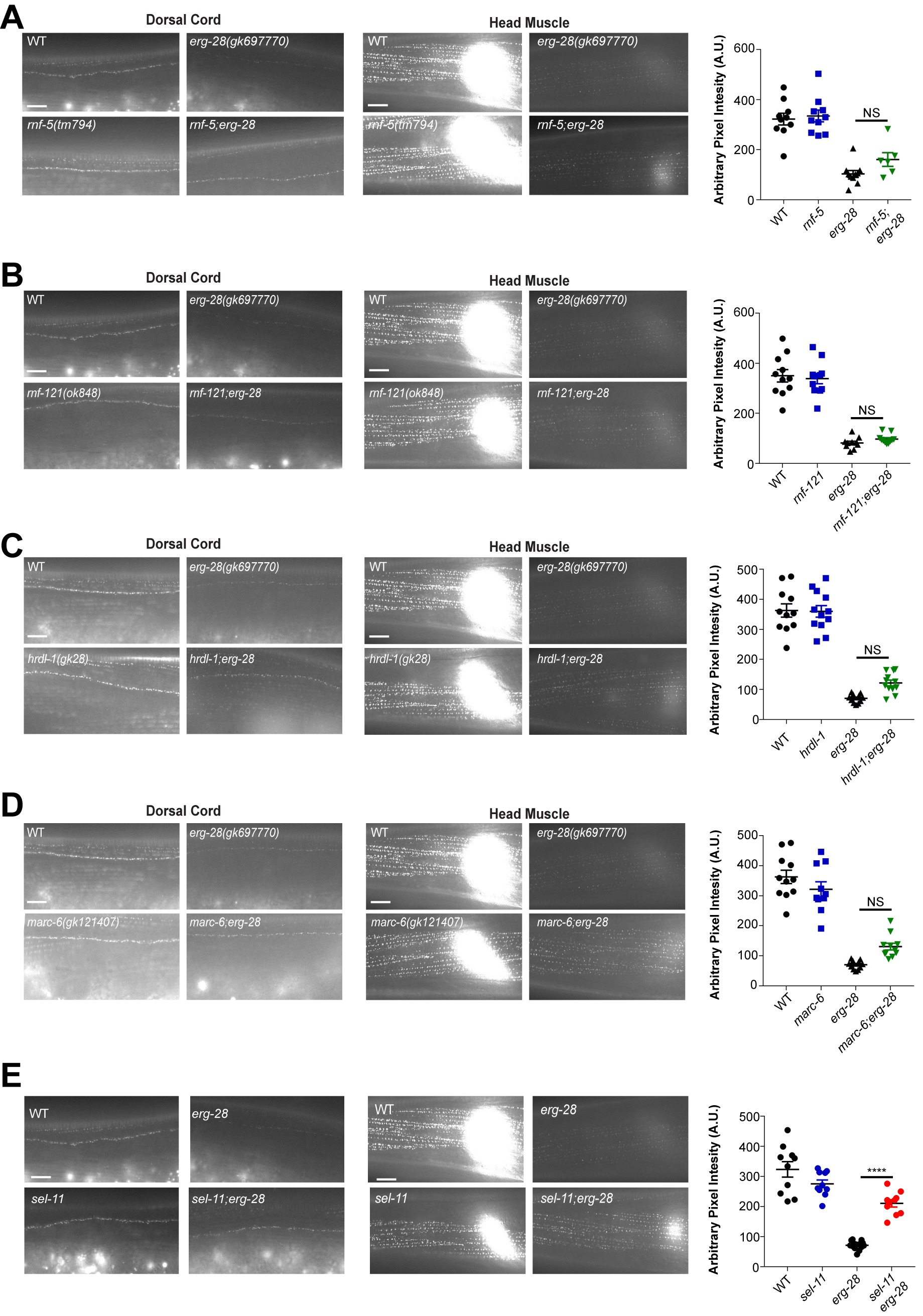

Supplement: S1 Fig — Representative images of SLO-1::GFP at the dorsal cords and head muscles, and quantification of SLO-1 at the dorsal cords. (A) rnf-5, (B) rnf-121, (C) hrdl-1, (D) marc-6, and (E) sel-11 mutations were screened for SLO-1 recovery in the erg-28 background. sel-11 mutation showed the most robust SLO-1 recovery. ****P<0.0001, NS, not significant, One-way ANOVA, Tukey’s post hoc test. (scale bar = 10 μm). (TIF) [file pgen.1008829.s001.tif]

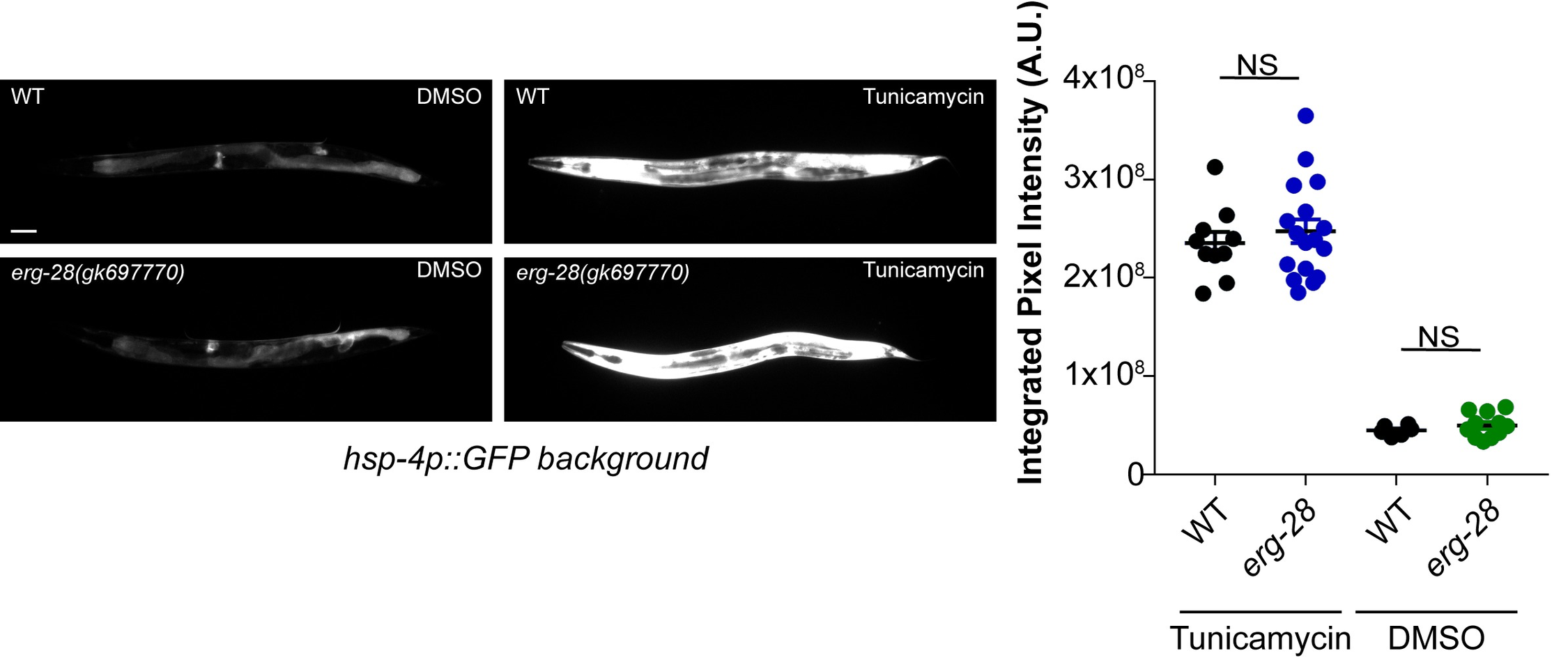

Supplement: S2 Fig — Representative images and quantification of the ER stress reporter hsp-4p::gfp expression in WT and erg-28 animals when treated with tunicamycin, a known ER stress inducer. Data are means ± SEM; NS, not significant, One-way ANOVA; Tukey’s post hoc test). (scale bar = 10 μm). (TIF) [file pgen.1008829.s002.tif]

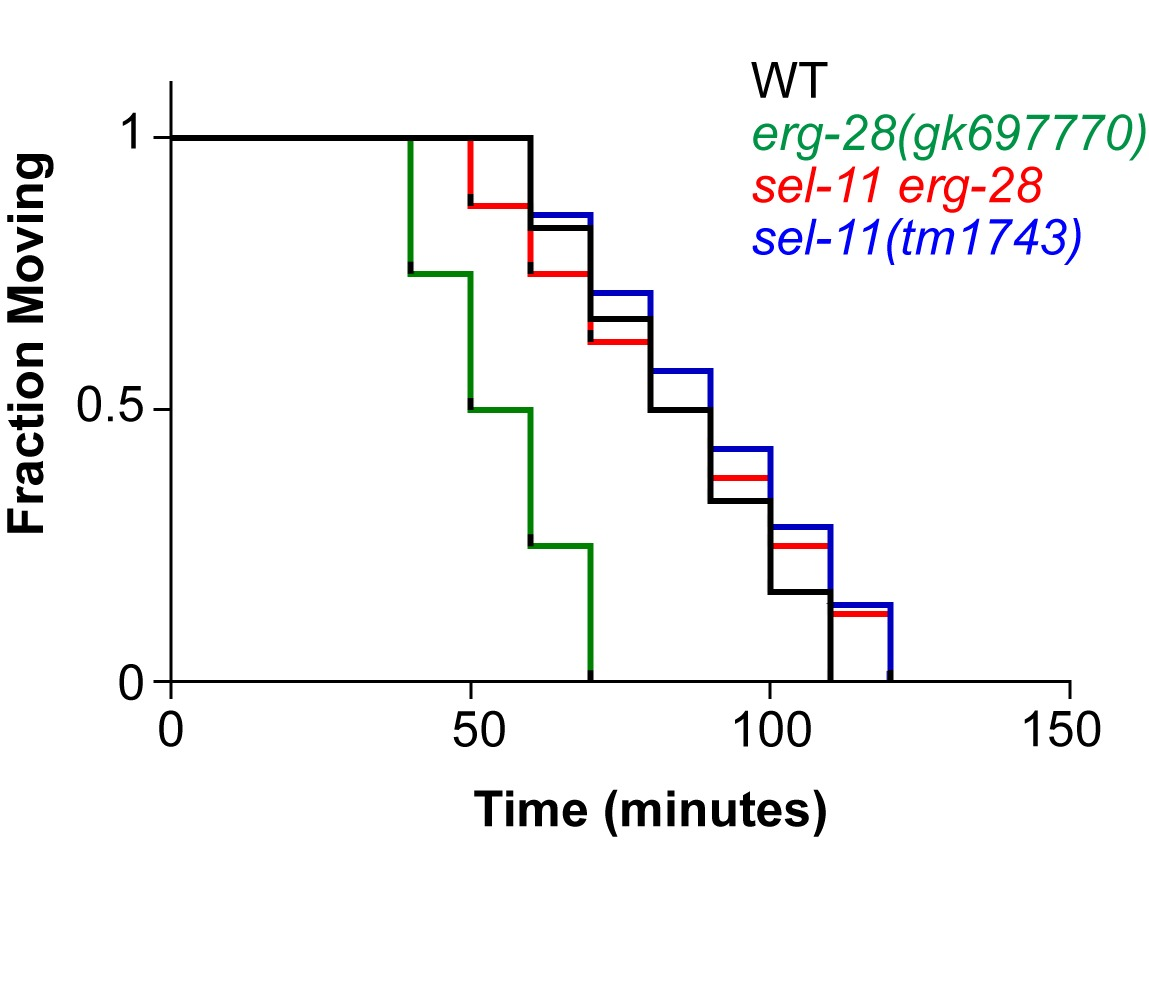

Supplement: S3 Fig — A sel-11 mutation increases aldicarb resistance in erg-28 animals. Aldicarb-induced paralysis was analyzed using Kaplan-Meier survival analysis. (TIF) [file pgen.1008829.s003.tif]

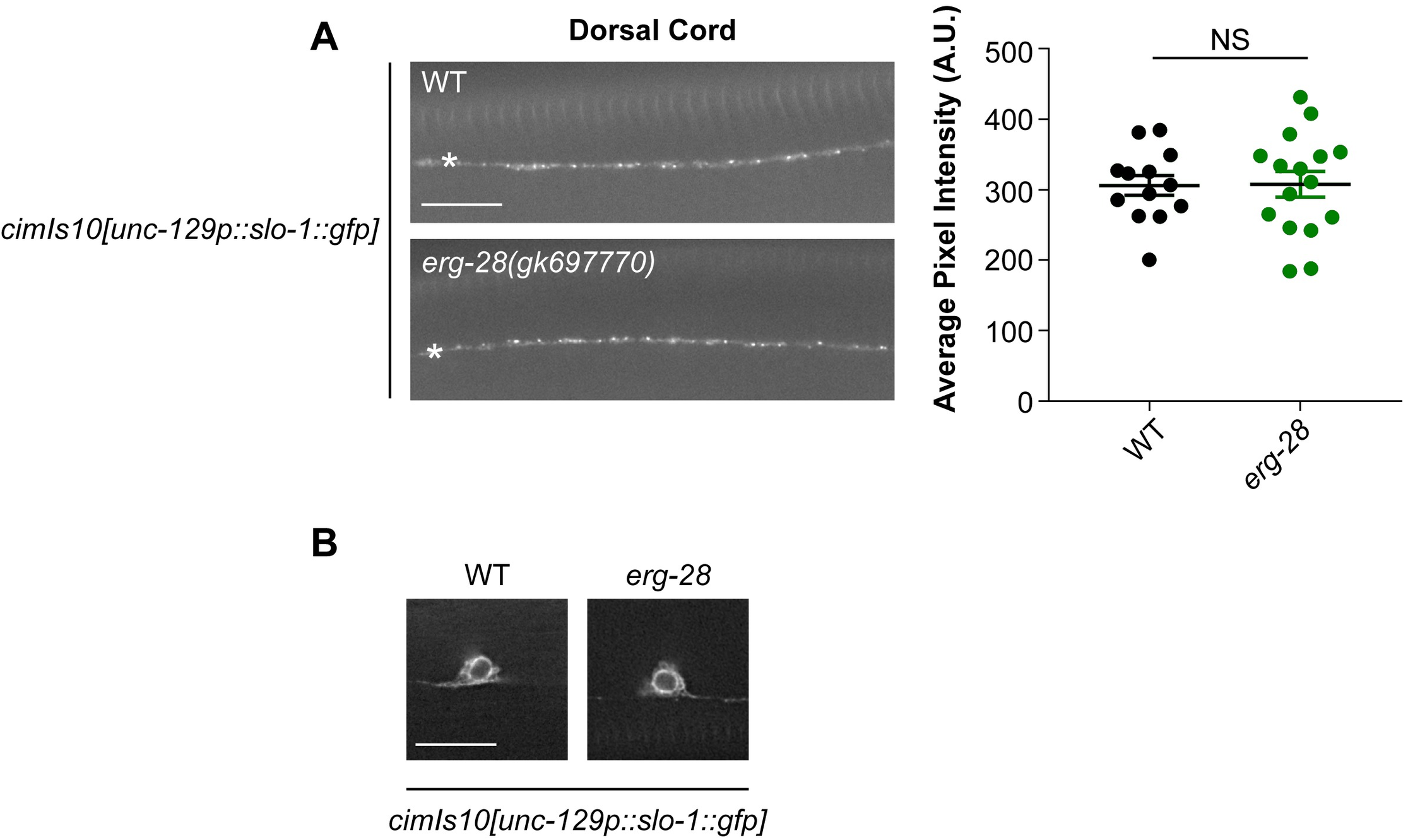

Supplement: S4 Fig — (A) Representative images and quantification of SLO-1 at the dorsal cord of cimIs10, a transgene that overexpresses SLO-1::GFP in DA and DB motor neurons. Data are means ± SE; NS, unpaired two-tailed t-test. (B) Representative images of SLO-1::GFP accumulated in the ER of both wild-type and erg-28 mutant animals. No aggregated puncta were observed (scale bar = 10 μm). (TIF) [file pgen.1008829.s004.tif]

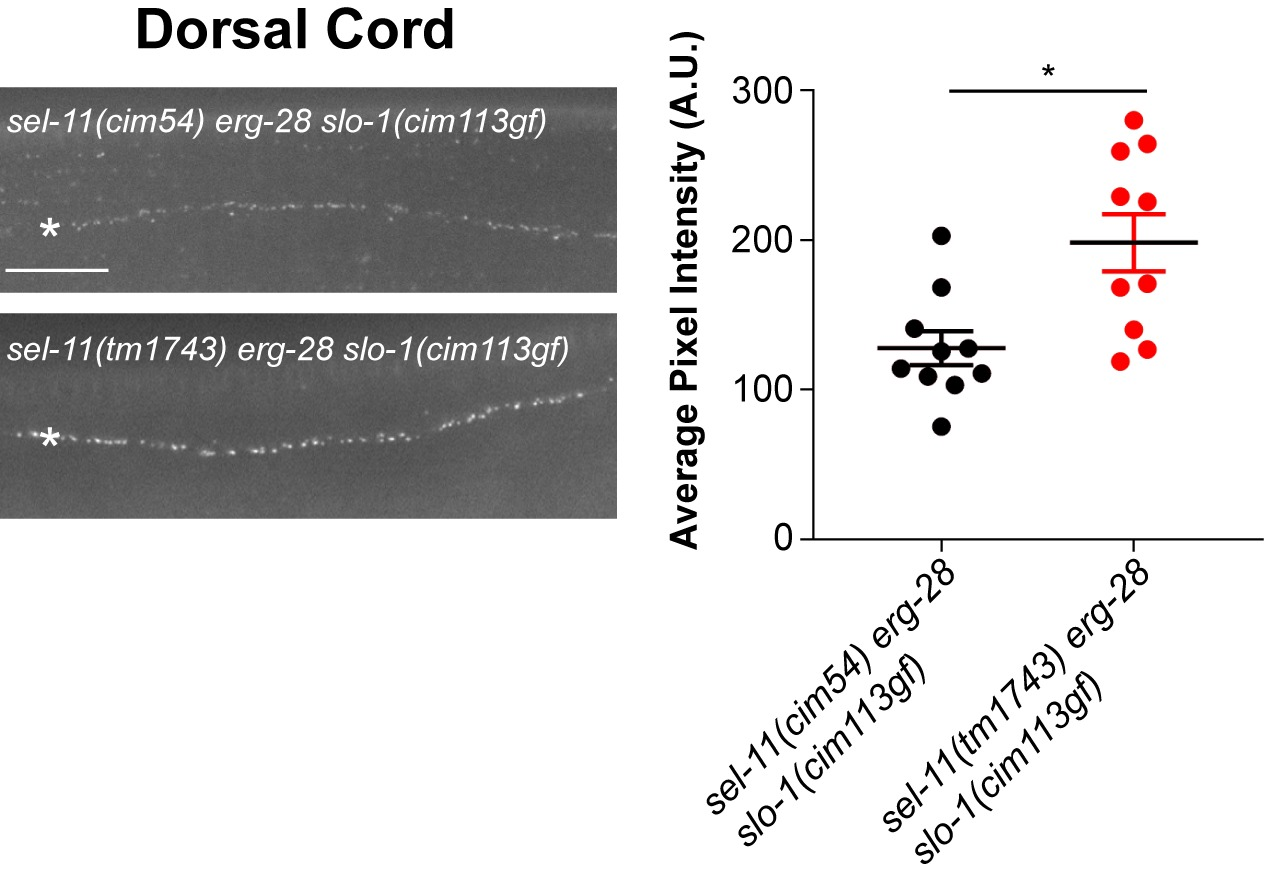

Supplement: S5 Fig — Representative images and quantification of SLO-1 at the dorsal cord of sel-11(tm1743) erg-28 and sel-11(cim54) erg-28. Data are means ± SEM; *P<0.05, paired two-tailed t-test. (scale bar = 10 μm). (TIF) [file pgen.1008829.s005.tif]

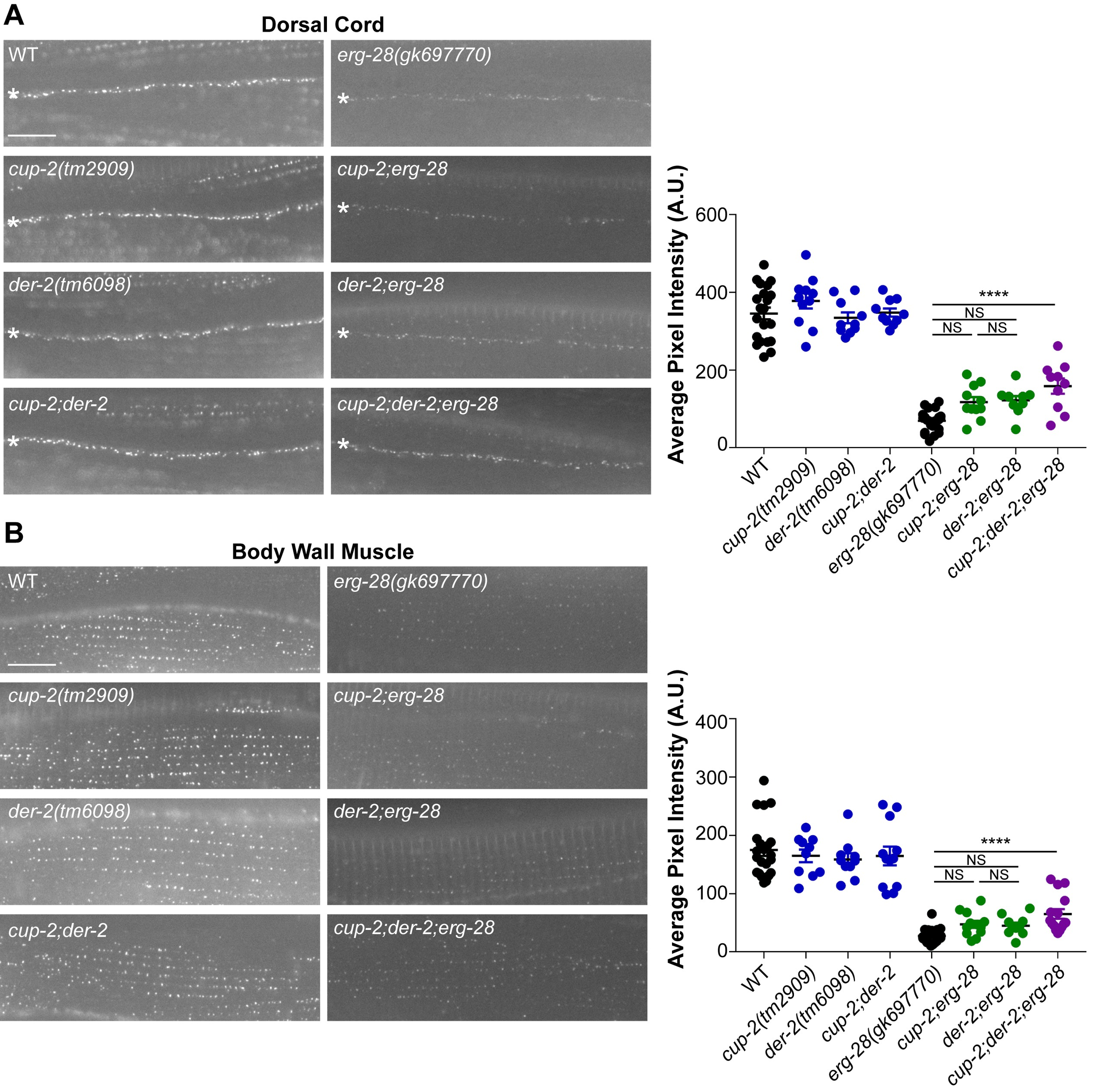

Supplement: S6 Fig — (A) and (B) Representative images and quantification of SLO-1 at the dorsal cord and body wall muscle. Individual Derlin mutations did not affect SLO-1 levels, but cup-2;der-2 double mutation elevated SLO-1 levels in an erg-28 background. Data are means ± SEM; ****P<0.0001; NS, not significant, One-way ANOVA, Tukey’s post hoc test. (scale bar = 10 μm). (TIF) [file pgen.1008829.s006.tif]

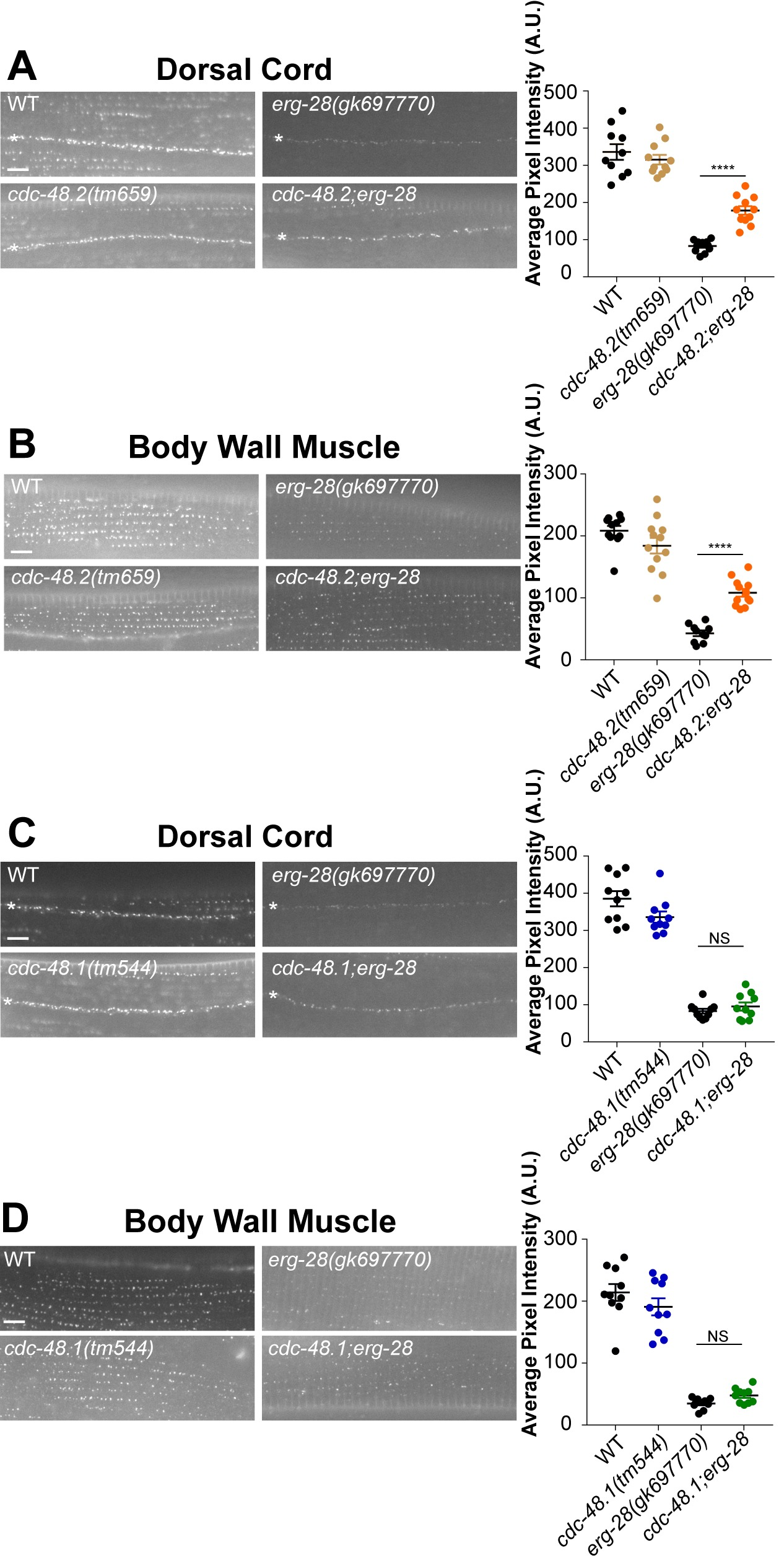

Supplement: S7 Fig — (A) and (B) Representative images and quantification of SLO-1 at the dorsal cord and body wall muscle. cdc-48.2 mutation elevated SLO-1 levels in erg-28 mutants. (C) and (D) Representative images and quantification of SLO-1 at the dorsal cord and body wall muscle. cdc-48.1 mutation did not elevate SLO-1 levels in erg-28 mutants. Data are means ± SEM; ****P < 0.0001, One-way ANOVA, Tukey’s post hoc test. (scale bar = 10 μm). (TIF) [file pgen.1008829.s007.tif]

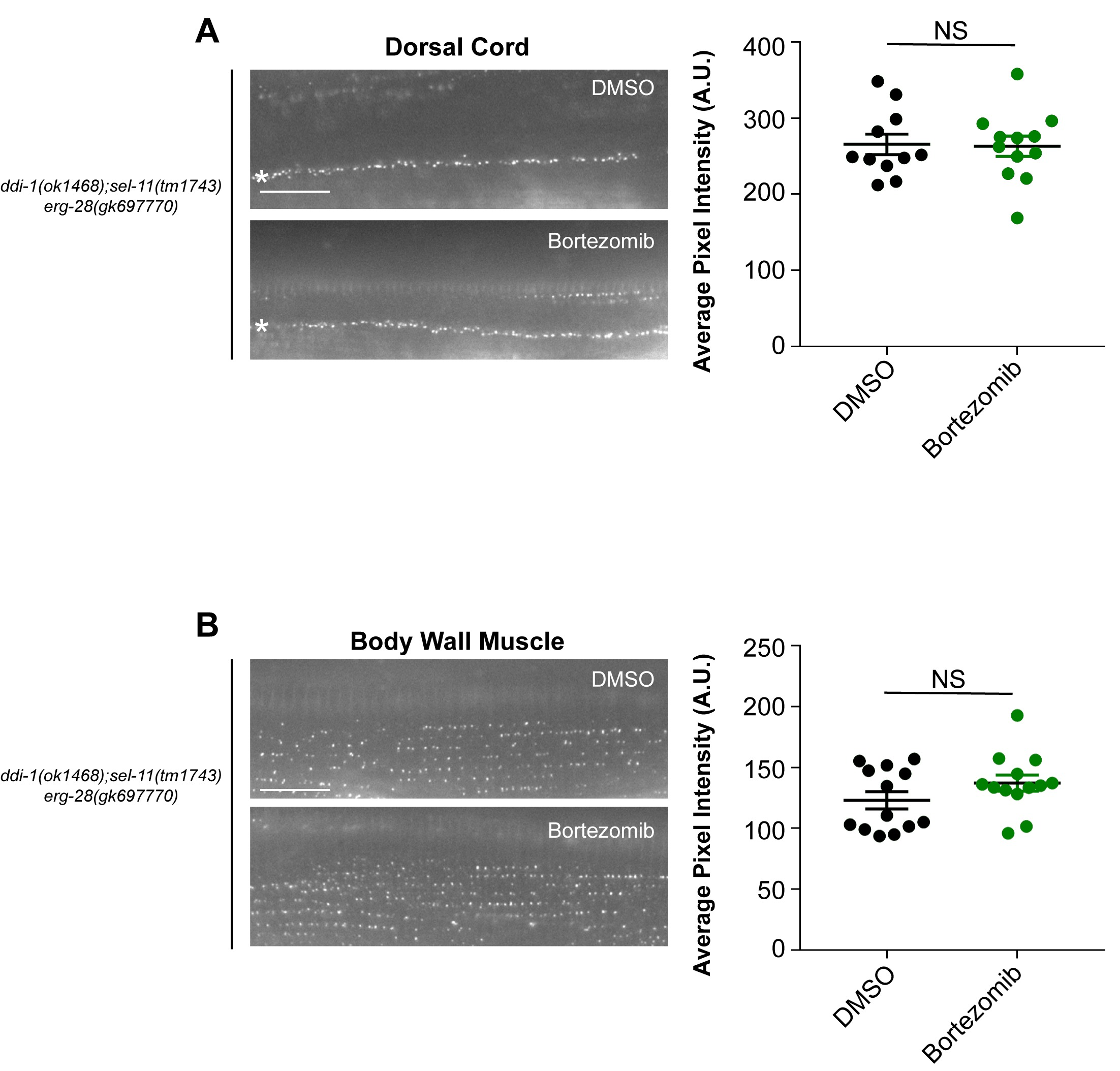

Supplement: S8 Fig — (A) and (B) Representative images and quantification of SLO-1 at the dorsal cord and body wall muscle when treated with 40 μM bortezomib, a proteasome inhibitor. Data are means ± SE; NS, unpaired two-tailed t-test. (scale bar = 10 μm). (TIF) [file pgen.1008829.s008.tif]

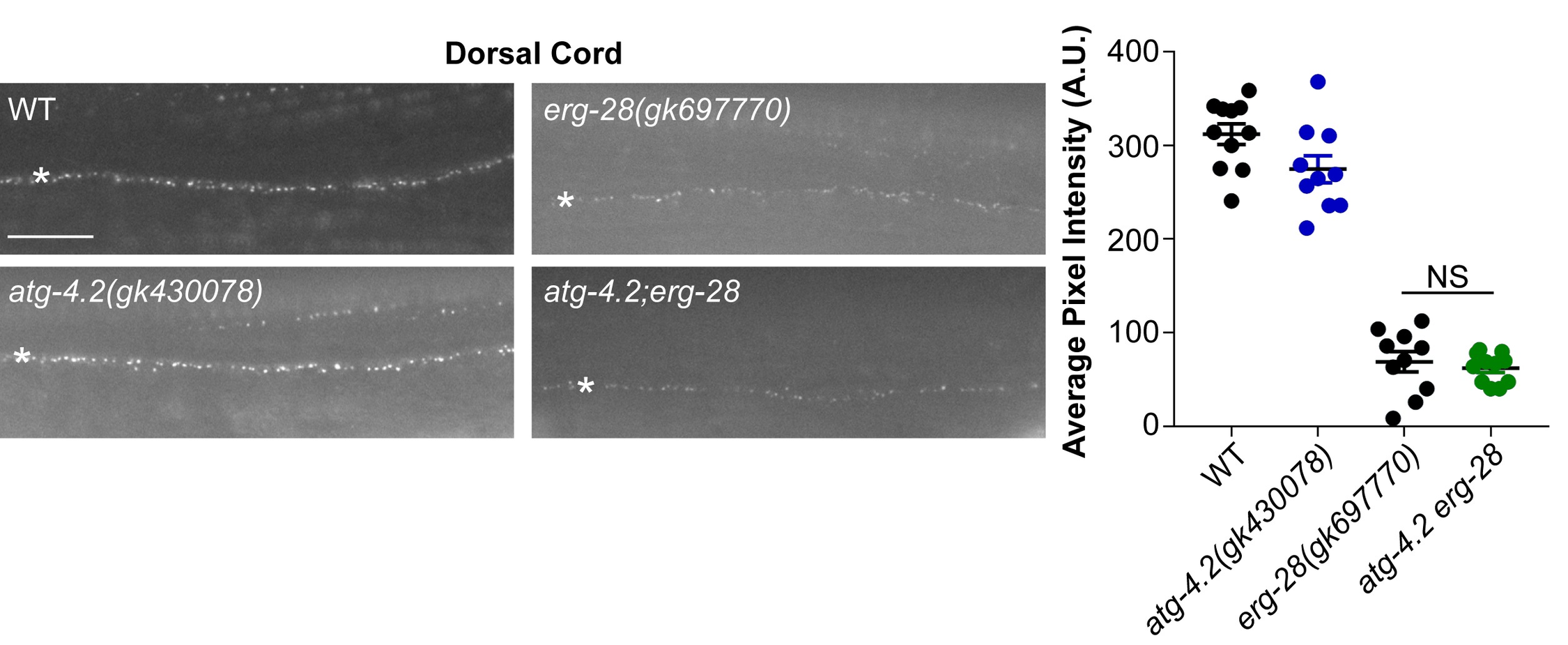

Supplement: S9 Fig — Representative images and quantification of SLO-1 in the dorsal cord (indicated by white asterisk). Data are means ± SEM; NS, not significant, One-way ANOVA, Tukey’s post hoc test. (scale bar = 10 μm). (TIF) [file pgen.1008829.s009.tif]

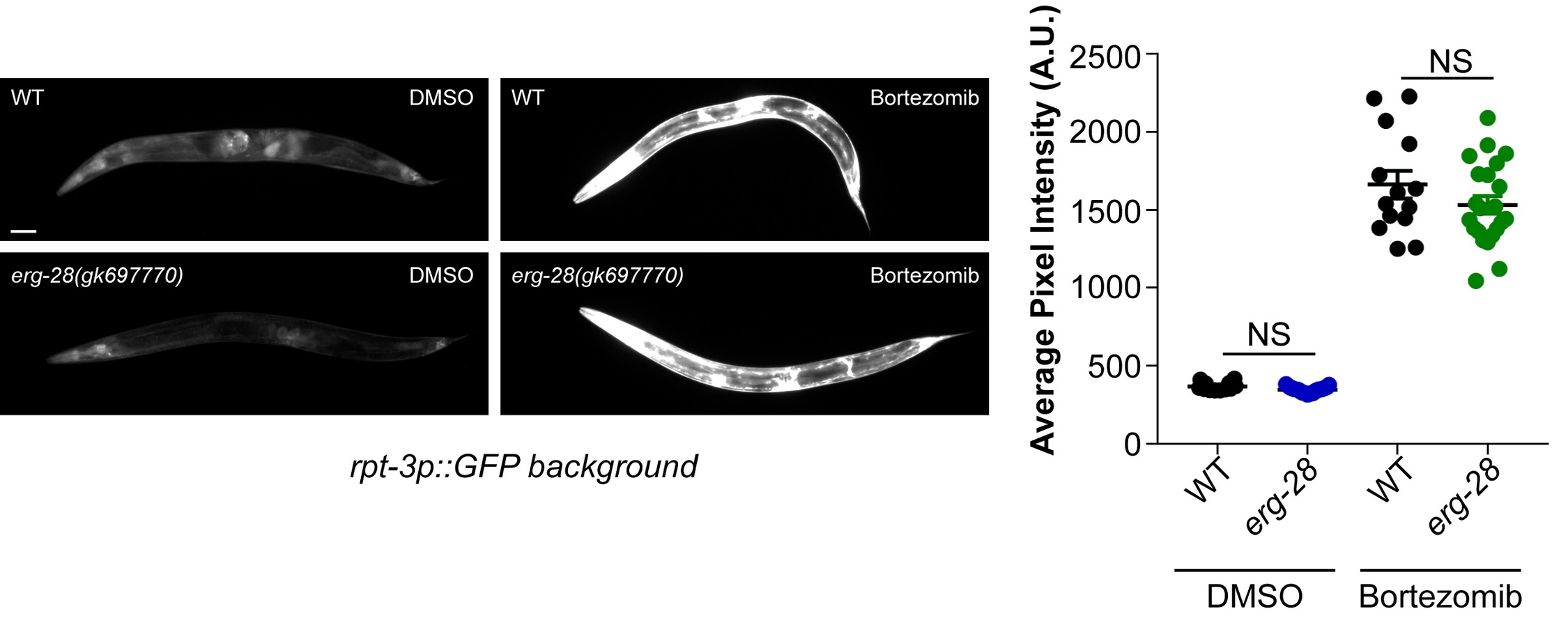

Supplement: S10 Fig — Representative images and quantification of rpt-3p::gfp expression in WT and erg-28 animals when treated with bortezomib, a proteasome inhibitor. Data are means ± SEM; NS, not significant, One-way ANOVA; Tukey’s post hoc test). (scale bar = 10 μm). (TIF) [file pgen.1008829.s010.tif]

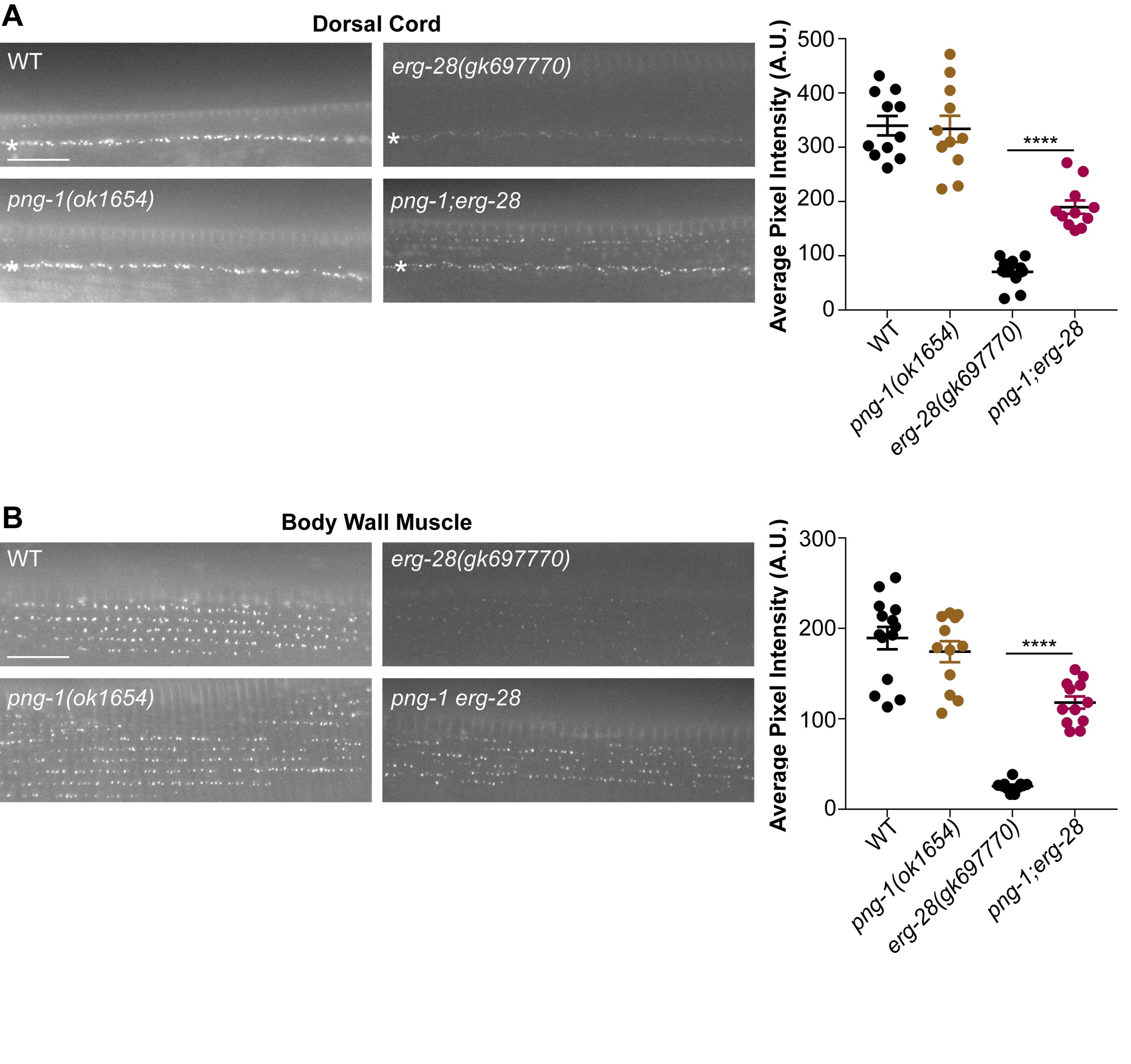

Supplement: S11 Fig — (A) and (B) Representative images and quantification of SLO-1 at the dorsal cord and body wall muscle. Data are means ± SEM; ****P < 0.0001, One-way ANOVA, Tukey’s post hoc test. (scale bar = 10 μm). (TIF) [file pgen.1008829.s011.tif]

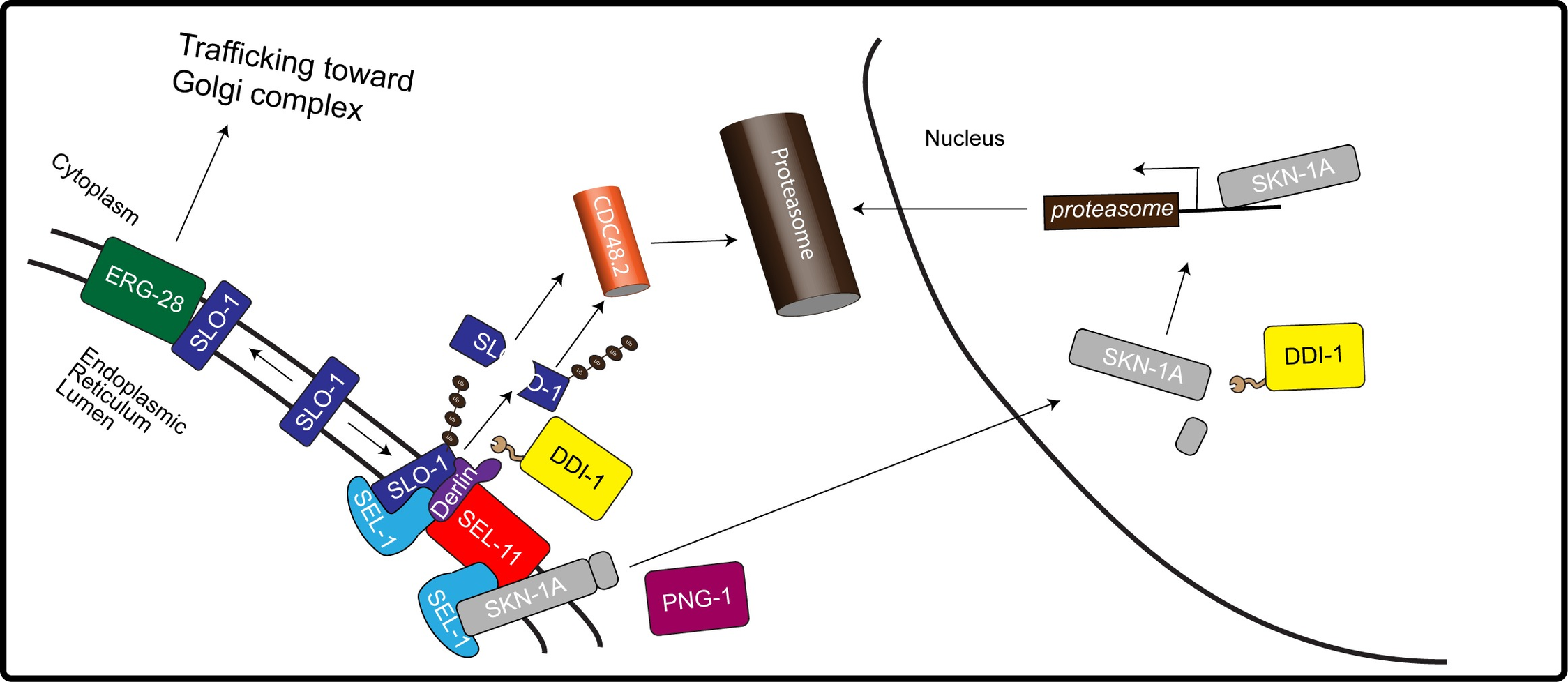

Supplement: S12 Fig — SLO-1 channels are normally trafficked to the Golgi complex with the ER membrane protein ERG-28. In the absence of ERG-28, SLO-1 channels are preferentially targeted for degradation by the SEL-11 E3 ubiquitin ligase complex, which consists of SEL-11/HRD1, SEL-1/HRD3, and Derlin homologs (CUP-2 and DER-2). Ubiquitination of SLO-1 signals extraction from the ER by the CDC-48.2/p97 unfoldase. The aspartic protease DDI-1 cleaves ubiquitinated SLO-1 channels either upstream or downstream of CDC-48.2 to facilitate extraction or proteasomal degradation. Partially degraded SLO-1 channels are ultimately degraded by proteasome. Additionally, a defect in the SEL-11 E3 ubiquitin ligase complex and DDI-1 reduces the overall level of proteasomes by blocking proper processing of the SKN-1A transcription factor, thus indirectly inhibiting SLO-1 ERAD. (TIF) [file pgen.1008829.s012.tif]
