# Supplementary material for: BK channel density is regulated by endoplasmic reticulum associated degradation and influenced by the SKN-1A/NRF1 transcription factor
Source: PLoS Genet. 2020 Jun 5;16(6):e1008829. doi: 10.1371/journal.pgen.1008829 (PMC7299407; doi:10.1371/journal.pgen.1008829)
Supplement: S1 Table — (PDF) [file pgen.1008829.s013.pdf]

S1 Table. *C. elegans* strains used.

Strain	Genotype	Source
**HKK796**	*slo-1(cim105)*	*Oh et al, 2017*
**HKK838**	*erg-28(gk697770) slo-1(cim105)*	*Oh et al, 2017*
**FX01743**	*sel-11(tm1743)*	National Bioresource Project of Japan
**HKK1203**	*sel-11(tm1743) slo-1(cim105)*	This Paper
**HKK1304**	*sel-11(tm1743) erg-28(gk697770) slo-1(cim105)*	This Paper
**FX04027**	*sel-1(tm4027)*	National Bioresource Project of Japan
**HKK1265**	*sel-1(tm4027) slo-1(cim105)*	This Paper
**HKK1266**	*sel-1(cim115) erg-28(gk697770) slo-1(cim105)*	This Paper
**FX794**	*rnf-5(tm794)*	CGC
**HKK1094**	*rnf-5(tm794);slo-1(cim105)*	This Paper
**HKK1415**	*rnf-5(tm794);erg-28(gk697770) slo-1(cim105)*	This Paper
**RB953**	*rnf-121(ok848)*	CGC
**HKK1416**	*rnf-121(ok848);slo-1(cim105)*	This Paper
**HKK1417**	*rnf-121(ok848);erg-28(gk697770) slo-1(cim105)*	This Paper
**VC20284**	*marc-6(gk121407)*	The Million Mutation Project
**HKK1070**	*marc-6(gk121407);slo-1(cim105)*	This Paper
**HKK1071**	*marc-6(gk121407);erg-28(gk697770) slo-1(cim105)*	This Paper
**VC35**	*hrdl-1(gk28)*	CGC
**HKK1095**	*hrdl-1(gk28);slo-1(cim105)*	This Paper
**HKK1418**	*hrdl-1(gk28);erg-28(gk697770) slo-1(cim105)*	This Paper
**FX02909**	*cup-2(tm2909)*	National Bioresource Project of Japan
**HKK1267**	*cup-2(tm2909); slo-1(cim105)*	This Paper
**HKK1183**	*cup-2(tm2909); erg-28(gk697770) slo-1(cim105)*	This Paper
**FX06098**	*der-2(tm6098)*	National Bioresource Project of Japan

**HKK1221**	*der-2(tm6098); slo-1(cim105)*	This Paper
**HKK1271**	*cup-2(tm2909); der-2(tm6098); slo-1(cim105)*	This Paper
**HKK1272**	*cup-2(tm2909); der-2(tm6098); erg-28(gk697770) slo-1(cim105)*	This Paper
**HKK1222**	*der-2(tm6098); erg-28(gk697770) slo-1(cim105)*	This Paper
**FX544**	*cdc-48.1(tm544)*	CGC
**HKK1308**	*cdc-48.1(tm544); slo-1(cim105)*	This Paper
**HKK1408**	*cdc-48.1(tm544); erg-28(gk697770) slo-1(cim105)*	This Paper
**FX5659**	*cdc-48.2(tm659)*	CGC
**HKK1309**	*cdc-48.2(tm659); slo-1(cim105)*	This Paper
**HKK1310**	*cdc-48.2(tm659); erg-28(gk697770) slo-1(cim105)*	This Paper
**RB1339**	*ddi-1(ok1468)*	CGC
**HKK979**	*ddi-1(ok1468); slo-1(cim105)*	*Oh et al, 2017*
**HKK980**	*ddi-1(ok1468); erg-28(gk697770) slo-1(cim105)*	*Oh et al, 2017*
**GR2203**	*ddi-1(mg572)*	CGC
**HKK1305**	*ddi-1(mg572); slo-1(cim105)*	This Paper
**HKK1306**	*ddi-1(mg572); erg-28(gk697770) slo-1(cim105)*	This Paper
**HKK1307**	*ddi-1(ok1468); sel-11(tm1743) erg-28(gk697770) slo-1(cim105)*	This Paper
**BZ142**	*slo-1(eg142)*	Davies et al,
**HKK940**	*slo-1(ky399)*	Davies et al,
**HKK552**	*erg-28(gk697770) slo-1(ky399)*	*Oh et al, 2017*
**HKK1237**	*sel-11(tm1743) slo-1(ky399)*	This Paper
**HKK1204**	*sel-11(tm1743) erg-28(gk697770) slo-1(ky399)*	This Paper
**RB1452**	*png-1(ok1654)*	CGC
**HKK1421**	*png-1(ok1654);slo-1(cim105)*	This Paper
**HKK1422**	*png-1(ok1654);erg-28(gk697770) slo-1(cim105)*	This Paper
**GR2183**	*mgIs72[rpt-3p::GFP+dpy-5(+)]*	CGC
**HKK1481**	*cimSi4[rgef-1p::skn-1a(cut,4ND)::mscarlet];mgIs72[rpt-3p::GFP+dpy-5(+)]*	This Paper
**SJ4005**	*zcIs4[hsp-4p::gfp]*	CGC
**HKK1520**	*erg-28;hsp-4p::gfp*	This Paper
**HKK238**	*cimIs10[unc-129p::slo-1::gfp]*	This Paper

**HKK521**	*erg-28(gk697770);cimIs10[unc-129p::slo-1::gfp]*	This Paper
**HKK1225**	*sel-11(cim54) erg-28(gk697770) slo-1(cim113gf)*	This Paper
**HKK1199**	*slo-1(cim113gf)*	This Paper
**HKK1200**	*erg-28(gk697770) slo-1(cim113gf)*	This Paper
**HKK1521**	*sel-11(tm1743) erg-28(gk697770) slo-1(cim113gf)*	This Paper
**VC39172**	*atg-4.2(gk430078)*	CGC
**HKK1523**	*atg-4.2(gk430078) slo-1(cim105)*	CGC
**HKK1522**	*atg-4.2(gk430078) erg-28(gk697770) slo-1(cim105)*	CGC
**HKK1353**	*erg-28(gk697770);mgIs72[rpt-3p::GFP+dpy-5(+)]*	This Paper
**HKK1402**	*sel-11(cim54) erg-28(gk697770) slo-1(cim113gf);cimEx107[sel-11 fosmid]*	This Paper
**GR2245**	*skn-1a(mg570)*	CGC
**HKK1087**	*skn-1a(mg570);slo-1(cim105)*	This Paper
**HKK1315**	*skn-1a(mg570);erg-28(gk697770) slo-1(cim105)*	This Paper
**HKK1354**	*ddi-1(ok1468);sel-11(tm1743) erg-28(gk697770) slo-1(cim105);cimEx108[rgef-1p::skn-1a[cut,4ND])*	This Paper
**HKK1359**	*cimSi4(rgef-1p::skn-1a(cut,4ND)::mscarlet]*	This Paper
**HKK1406**	*cimSi4[rgef-1p::skn-1a[cut,4ND]::mscarlet];sel-11(tm1743) erg-28 (gk697770) slo-1(cim105)*	This Paper
**N2**		CGC
