# Supplementary material for: BK channel density is regulated by endoplasmic reticulum associated degradation and influenced by the SKN-1A/NRF1 transcription factor
Source: PLoS Genet. 2020 Jun 5;16(6):e1008829. doi: 10.1371/journal.pgen.1008829 (PMC7299407; doi:10.1371/journal.pgen.1008829)
Supplement: Attachment — Submitted filename: 04132020_TPC Response_to_reviewers-1.pdf [file pgen.1008829.s015.pdf]

Response to the reviewers' comments:

We appreciate the reviewers for the thorough, insightful comments on our work. Based on these comments, we have completed additional experiments and clarified the concerns raised by the reviewers. We believe that this additional work has made our manuscript stronger.

All three reviewers raised a concern on whether SLO-1::GFP is normally folded or misfolded in the *erg-28* mutant. This concern largely results from our wrong wording "chaperone" in the abstract. We corrected it and further clarified in the manuscript with additional experiments.

The core of the concern is whether ERG-28 is a chaperone or not. Our published study indicates that ERG-28 is not an ER chaperone, but an ER membrane protein that aids the exit from the ER (Oh et al 2017). Our previous study showed that unlike ER chaperones, ERG-28 is a highly mobile protein that shuttles between the ER and the ER-Golgi intermediate compartment (ERGIC), evidenced by the critical role of its C-terminal COPI binding site. Therefore, it is likely that SLO-1 channel trafficking is delayed in the *erg-28* mutant, and SLO-1 channels are recognized as misfolded even though they are not.

Once proteins become misfolded, they will adopt a particular energetically stable conformation or become aggregated together. Such misfolded or aggregated proteins cannot be correctly refolded without the aid of a chaperone and the expense of energy. If SLO-1 channels become misfolded in the absence of ERG-28, the further absence of the ER E3 ubiquitin ligase SEL-11 will not cause refolding.

In addition, we performed additional experiments, based on the idea that in the absence of ERG-28 overexpressed SLO-1 channels will not undergo trafficking beyond the ER and thus cannot incorporate into SLO-1 puncta at presynaptic terminals if SLO-1 channels are not normally folded. We used an integrated transgene to overexpress GFP-tagged SLO-1 in a subset of motor neurons and examined their levels at the presynaptic terminals of the dorsal cord of wild-type and *erg-28* mutant animals (line: 214, S4 Fig). We did not find any significant difference in SLO-1 levels of the dorsal cord between wild-type and *erg-28* mutant animals, supporting our conclusion that SLO-1 channels are normally folded in *erg-28* mutant animals.

Our study is based on *C. elegans* genetic analysis. Given the low expression levels of SLO-1 channels in limited tissues (relying on the endogenous promoter), biochemical study to measure folding or misfolding is not possible to perform. Based on the reviewers' comments, we toned down our conclusions and discussed alternative possibilities.

All of the changes we made in the revision were written in blue. We have included further a point-by point response to each of the reviewer's comments.

Reviewer #1: *Cheung et al present a nice study using C. elegans to determine that BK potassium channel density is regulated by ERAD and proteasome degradation via the transcription factor SKN-1. All of the molecules studied have orthologs in mammals. Their epistasis analyses using behavioral, physiological and GFP-tagged molecules in nervous system and muscle present a mostly clear story on how levels of the BK channel SLO-1 appears to be regulated from the ER to the Golgi. Impressively, the authors worked hard to find genes involved in this process via reserve and forward genetics, and along the way they generated many double, triple mutants with many genes inconveniently located on the same chromosome. Additionally, they introduced a variety of difficult-to-generate transgenetic strains. This includes (1) a CRISPR knock-in of a gain-of-function mutation in the endogenous slo-1 gene that was already tagged in frame with gfp, (2) a miniMos knock-in of an activated form of skn-1a that predicted to keep the proteasome extra active. I feel that the writing is mostly clear and compliment them on a well-done study.*

Major issues:
*1. I'm confused about whether you think that normal, correctly folded SLO-1 is subject to this form of degradation, and/or only misfolded versions of SLO-1. Do you think that the *delay* in trafficking of SLO-1 in the erg-28 mutant itself leads to a misfolding of SLO-1? And therefore, the misfolding might be detected and use SEL-11 to direct it to the proteasome? Do you think that the gf mutant version of SLO-1 chosen is subject to misfolding more than WT version? If so, how might this effect your interpretation of results?*
Our current data support that correctly folded SLO-1 is subjected to SEL-11-mediated degradation. Once proteins become misfolded, they will adopt a certain energetically stable conformation or aggregate together. Such misfolded or aggregated proteins cannot be correctly refolded without the aid of a chaperone and the expense of energy. If SLO-1 channels become misfolded in the absence of ERG-28, the further absence of the ER E3 ubiquitin ligase SEL-11 will not cause refolding. The fact that we can observe a large amount of SLO-1 channels at presynaptic terminals and muscle excitation sites in the *sel-11 erg-28* double mutant in comparison to the *erg-28* mutant indicates that SLO-1 channels in the absence of ERG-28 are not misfolded.
We agree that the delay in trafficking SLO-1 in the *erg-28* mutant is responsible for SLO-1 degradation. The role of ERG-28 in trafficking was previously demonstrated [ERG-28 protein is a highly mobile protein (Oh et al, 2017), which is consistent with functional importance of its COPI binding motif. This suggests that ERG-28 is likely involved in the transport of SLO-1 channels to the Golgi complex]. However, because we can reverse SLO-1 degradation by *sel-11* mutation, it is likely that the delay in trafficking signals the ERAD and/or quality control machinery to consider SLO-1 channels misfolded.

It is possible that certain gain-of-function mutations can cause a protein misfolding and result in a lower amount of proteins even in the wild-type background. While we cannot evaluate the SLO-1 levels in the *ky399* allele, the levels of SLO-1(*cim113gf*), which is

GFP- tagged, are comparable to wild-type SLO-1(*cim105*) levels. This result is now presented in Fig 3A. Due to potential complications of SLO-1(gf), we always confirm the results with wild-type SLO-1(cim105).

2. Starting on pg 12 and in figure 5, you deduce that ddi-1 and sel-11 work in series to elevate SLO-1::gfp in erg-28 since combining them does not elevate higher than either single mutant. However, how do you know that SLO-1::gfp levels at a ceiling that would prevent you from seeing additive effects here? Same issue for testing together with the proteasome inhibitor bortezomib.

The levels of SLO-1::GFP in *erg-28;ddi-1;sel-11* mutants did not go up to wild-type levels of SLO-1::GFP. This indicates that a ceiling effect the reviewer was referring to is not present.

3. The novel single-gene knock-in transgenic worm carrying pan-neuronal cimSi4, Prgef-1::skn-1a[cut, 4ND], seems very useful as a way to upregulate proteasome function. Although the present results are consistent with it upregulating not only proteasome expression but also activity, it would be safe to test this idea independently.

The ability of *skn-1a[cut, 4ND]* increases proteasome activity was previously shown by Lehrbach et al. (2019). Lehrbach et al. used a strain expressing Ub(G76V)::GFP. The point mutation of Ub(G76V) targets ubiquitin to be constitutively degraded. We repeated the same experiment with *cimSi4[rgef-1p::skn-1a(cut, 4ND)]* transgene. As reported, *mgIs77[ub(G76V)::gfp]* exhibits increased GFP levels in the *skn-1a(mg570)* background compared to wild-type. Introduction of the *cimSi4* transgene into *skn-1a;mgIs77[ub(G76V)::gfp]* animals decreased Ub(G76V)::GFP levels, compared to *skn-1a;mgIs77[ub(G76V)::gfp]* animals. This data, in conjunction with the fact that *cimSi4* can induce degradation of SLO-1 in *sel-11 erg-28* animals, is consistent with the hypothesis that *cimSi4* transgene indeed upregulates proteasome activity as well as expression.

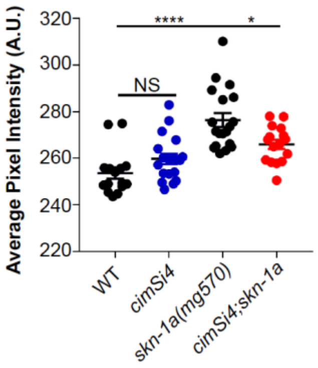

<u>*Minor issues:*</u>

1. You might consider replacing N2 with WT throughout, expect for Methods, so that people outside of worm field may better understand your work.

We appreciate the reviewer’s suggestion. We replaced N2 with WT throughout the main text.

2. You talk about a deletion mutant for sel-11 in Fig 1&2 and a distinct sel-11 mutant in Fig 3. This is confusing for readers. To help them understand, note the allele for each in all figures so they know which one is which. Also related, in panel Fig 2e, it is confusing to only note the allele cim54 without letting readers know it is an allele of sel-11. Please note sel-11(cim54) erg-28 here, without a semicolon to be consistent with the sel-11 erg-28 in Fig 1. It is convention to use a space rather than a semicolon for mutations on the same chromosome in C. elegans.

As requested by the reviewer, we clarified all of the issues related to the allele designation.

We added *sel-11* allele names to Fig 1-3. We clarified the allele *cim54* by replacing it with *sel-11(cim54)*. We removed the semicolon in Fig 1.

3. The title for Fig2 doesn't fit with the actual data on behavioral and physiological consequences for suppressing the slo-1(gf) phenotypes. These data do not directly demonstrate that SEL-11 targets SLO-1 for "degradation". They are consistent with this idea, but do not directly support it. Fig 2a and c, having + to represent slo-1(gf) single mutant is confusing, especially since it looks like an "x" when tilted. Fig 2b. there appears to be a light green line that does not match any of the colors in the key. It is also difficult to determine which line matches the slo-1(gf) single mutant. There has got to be a better coding scheme for these data.

In response to the reviewer's comments, we changed the title of Fig 2 and the coding scheme to be more obvious to readers. The title now reads: "A *sel-11* mutation reverses reduced SLO-1 functions observed in the absence of ERG-28."

4. In Fig 3a& b, it is hard to be convinced that SLO-1::gfp levels are normal in the gf allele due to saturated image. Perhaps you could include a colorized version of a non-saturated greyscale image to better show this finding? In Fig 3b I'm confused why you need arrows. They don't signify time or order, so eliminate. Fig 3c seems incomplete since it doesn't indicate mutagenized worms, and other aspects of the screen. Nowhere in Fig 3 do you describe what transgene is included on cimEx107, so its impossible to interpret Fig3e&f. For Fig 3g, it would be useful to highlight the area deleted by allele tm1743.

We quantified *slo-1::gfp* levels at the dorsal cord and body wall muscles of wild-type and gain-of-function mutant animals and added its results to Fig 3A. We found no significant difference when we compared *slo-1(cim113gf)* and *slo-1(cim105)* animals (Fig 3A). We eliminated the arrows in Fig 3B. We annotated the transgene *cimEx107[sel-11]*, in conjunction with the description of *cimEx107* in Fig 3 legend and Supplementary Table 1. We highlighted the area deleted by allele *sel-11(tm1743)*.

5. The photos focusing on the dorsal cord show GFP-tagged SLO-1 in the neurons but also in muscle nearby. Help the reader naïve to worms focus on the nerve cord with a bracket on the sides of all photos.

As the reviewer suggested, we added asterisks to all of the dorsal cord images.

6. Readers will not understand contents of cimEx108 for Fig 6. Why not replace with [skn-1a]? Or something similar? Likewise, think of a way to write cimSi4 more

informatively in the figures; you can probably skip noting the sel-11 and erg-28 alleles to save space here.
We annotated *cimEx108[skn-1a(cut,4ND)]* in Fig 6B and "*cimSi4[rgef-1p::skn-1a[cut,4ND]"* in Fig 6C. Further description of these transgenes is in Figure legends and Supplementary Table 1. To save space, we omitted the alleles of *sel-11* and *erg-28* in Fig 6D.

7. The figure legend of Fig S1 refers to Fig 1 that sel-11 suppressed decreased expression of SLO-1::gfp in erg-28 more than in other ER-resident E3 ubiquitin ligases, but it would be nice to see the sel-11 results repeated side by side with the sel-11 results in Fig S1 for easier comparison.
We provided a set of *sel-11* dorsal cord and head muscle images and quantified all of the dorsal cord images in S1 Fig.

8. Line 137. List all ER-resident E3 ubiquitin ligases tested here.
We listed all ER-resident E3 ubiquitin ligases tested in lines: 153-156.

9. Note "in vivo imaging" on line 150. These in vivo results are much more meaningful than any Western blot.
We agree with the reviewer and noted "in vivo fluorescent imaging" in line: 169.

10. Pg 10, line 250. You note the gene der-2; however, Wormbase does not list any gene by this name. Instead, it is currently named R151.6 and noted is an ortholog of human DERL2 (derlin 2). Please list the appropriate gene name so that others can follow along with Wormbase. Or get Wormbase to change the name of the gene before you publish your paper.
We communicated with Dr. Tim Schedl and WormBase, and they agreed to change the name of the gene *R151.6* to *der-2*.

Reviewer #2: *In the manuscript "BK channel density is regulated by endoplasmic reticulum associated degradation and influenced by the SKN-1A/NRF1 transcription factor", the authors employ genetic analyses to identify key players in the degradation of the SLO-1 channel in C. elegans. The authors use a candidate gene approach to demonstrate that the E3 ubiquitin ligase, SEL-11, is responsible for SLO-1 degradation in erg-28 mutant animals. Furthermore, using an unbiased forward genetic screen the authors identified sel-11 as a gene involved in SLO-1 degradation. Next, the authors continue to elegantly and carefully test candidate genes in the ERAD degradation pathway and propose a model where the efficiency of ER trafficking balanced with ER degradation affects density of functional SLO-1 levels at the presynaptic axon terminal.*

The manuscript is a solid body of work that contributes to our understanding of BK channel density at the cell surface. Alterations in channel density impact a plethora of functions such as synaptic transmission, secretion, and hormone release. Changes in functionality or distribution of these voltage calcium activated potassium channels are associated with multiple diseases such as alcoholism, epilepsy and neurodegenerative diseases. This manuscript provides evidence of the susceptibility of BK channels to

ERAD. These findings have important implications for maintaining proper functionality of excitable cells. I found this work to be interesting, well executed, and well communicated and would be appropriate for publication in PLoS Genetics after addressing the issues outlined below.

<u>Major comments</u>

1. The authors' classification of ERG-28 as an ER chaperone is overreaching. This classification must be demonstrated by documenting changes in the conformation of SLO-1 in the absence of ERG-28. Turnover by ERAD can be a result of misfolding or in appropriate accumulation. The proper designation for ERG-28 an ER resident protein or ER membrane protein. Not an ER chaperone.

We agree with the reviewer's comments. In fact, our past and current data support that ERG-28 is an ER membrane protein that plays a role in SLO-1 trafficking. We now designate ERG-28 as an ER membrane protein.

2. The authors state that SLO-1 tagged GFP is normally folded based on the functional data demonstrating normal movement and aldicarb sensitivity (Oh et al 2017). With this in mind, the authors propose a role of ERAD in a normally folded SLO-1. However, the assays used to justify proper folding lack the sensitivity to make this claim. Experimental data (protease sensitivity, propensity for aggregation) is lacking to show folding is "normal".

Each experimental system has its own limitations. The classical experiments the reviewer stated are biochemical. These experiments are not perfect, although they directly deal with protein folding. For example, such experiments do not really address the functional aspect of proteins. In our genetic study, we do not overexpress GFP-tagged SLO-1, so it provides a unique opportunity to address the functional aspect of SLO-1 ERAD. However, we cannot perform biochemical experiments because the expression of SLO-1 is very low, tissue-specific, and limited to the membrane. Nonetheless, we performed additional experiments, based on the idea that overexpressed SLO-1 channels in the absence of ERG-28 will not undergo trafficking beyond the ER if SLO-1 channels are not normally folded. We used an integrated transgene to overexpress GFP-tagged SLO-1 in DA and DB motor neurons and examined their levels at the presynaptic terminals of the dorsal cord of wild-type and *erg-28* mutant animals (lines: 223-230, S4 Fig). We did not find any significant difference in SLO-1 levels of the dorsal cord between wild-type and *erg-28* mutant animals, supporting our conclusion that SLO-1 channels are normally folded in *erg-28* mutant animals. Additionally, we found that while SLO-1 channels accumulated in the ER of both wild-type and *erg-28* mutant animals, they were not aggregated puncta, which is a sign of misfolding.

<u>Minor comments</u>

1. In line 135, the authors state that they introduced null mutations in all the known ER-resident E3 ubiquitin ligases. However, they do not provide a reference or explanation as to how these candidates were identified or defined.

ER resident E3 ubiquitin ligases were chosen based on prior publications by others. We added references.

2. The authors conclude that SEL-11 is the "major" E3 ubiquitin ligase responsible for SLO-1 degradation in erg-28 mutant animals. Quantitative measures of pixel intensity for each of the other E3 ubiquitin ligase in Figure S1 are needed to demonstrate this conclusion (similar to quantification in Figure 1A and 1B, and in Fig S3-S6).
We added representative images of the dorsal cord and head muscle in the *sel-11* set of animals in S1 Fig S1. In addition, we included the quantification of SLO-1 in S1 Fig.

3. Quantification of SLO-1 levels in the Western in Figure 1C are needed to demonstrate that the sel-11 mutant "considerably restored" SLO-1 levels (Line 152). Is this difference significant?
We included quantification of SLO-1 levels in the Western in Fig 1C. We repeated the Western blot analysis and found similar results. We also mention that, in addition to Western blot analysis, *in vivo* fluorescent imaging and functional analysis, all together support the considerable restoration of SLO-1 levels.

4. In Figure 2, the color scheme in panel B makes it difficult to identify which curve matches which genotype in the key. Considering using fewer beige/brown/gold colors.
We changed the color scheme to clarify different genotypes.

5. In line 209, the authors state that the slo-1(cim113gf) animals show low-frequency thrashing in Figure 3E. However, the figure panel indicates the slo-1(ky399gf) allele is used. This is also the case in Figure 3D (with the GFP signal, should be cim113gf). Please review the figure panels and specify correct allele used in the experiment.
We appreciate the comment for pointing out the errors in labeling. We corrected the figure panel to indicate that the *slo-1(cim113gf)* allele was used in Fig 3D and 3E.

6. Quantification of the levels of SLO-1 rescue by sel-11(cim54) in Figure 3F is recommended. It would be interesting to compare the levels in the missense vs null allele (Figure 1C). This analysis may reveal whether this point mutation affect the overall stability of SEL-11 protein or if this point mutation does indeed impair the interaction with substrate SLO-1.
We quantified the levels of SLO-1 in the dorsal cords of *sel-11(cim54) erg-28 slo-1(cim113gf)* mutants and compared them to *sel-11(tm1743) erg-28 slo-1(cim113gf)* animals. The null mutation in the *tm1743* allele recovered higher levels of SLO-1 at the dorsal cord than *cim54*. We incorporated this new data into S5 Fig. Without further experiments, we believe that we cannot conclude whether the *cim54* mutation impacts SEL-11 structure or the interaction with SLO-1.

Reviewer #3: *Cheung et al. Manuscript Review*

This study shows that in the absence of the ER chaperone ERG-28, the BK channel SLO-1 is degraded via the SEL-11 ligase complex as part of ERAD, and in parallel via a pathway regulated by SKN-1A. More specifically, Cheung et al. show that in erg-28 mutants, SLO-1 protein levels in the dorsal cord and muscle are strongly reduced. This

reduction in SLO-1 protein is partially suppressed by mutants lacking components of an ER resident E3 ligase complex including SEL-11/HRD1, SEL-1/HRD3, CUP-2 and DER-2. ERAD is known to be mediated by SEL-11 and by the AAA-ATPase CDC-48. Interestingly, SLO-1 degradation requires CDC-48.2 but not CDC-48.1, which may be the first specific function for CDC-48.2. Based on these observations, the authors conclude that SLO-1 is degraded via ERAD in the absence of erg-28. The authors identified sel-11 as a regulator of SLO-1 based on two independent approaches: (i) a candidate screen of 5 ER resident E3 ligases, and (ii) a clever forward genetic screen that capitalized on the sluggish movement phenotype of a slo-1 gain of function mutant. The authors nicely show that erg-28 mutants suppress slo-1(gf) phenotypes including sluggish movement, resistance to paralysis by aldicarb, and reductions in evoked currents in muscle. This suppression is likely due to ERAD because the suppression of SLO-1 function can be reversed by mutation of the sel-11 ligase. These experiments suggest that the SLO-1(gf) channels are functional even in the absence of the ER chaperone erg-28. A major finding in this manuscript is that normally folded SLO-1 is surprisingly degraded via ERAD. While the genetic experiments are consistent with this idea, they do not provide definitive evidence that SLO-1(gf) channels are folded normally in the absence of the chaperone erg-28. The simplest explanation is that the ER chaperone erg-28 is required for normal folding or tetramer assembly of BK channels and in the absence of erg-28, the unfolded or misassembled SLO-1 subunits are targeted for ERAD. This interpretation is consistent with ERG-28 acting as a traditional chaperone for SLO-1. In this alternative model, inhibition of ERAD of SOL-1(gf) in sel-11 mutants gives misfolded SLO-1(gf) extra time to refold in the ER and to continue along the secretory pathway to the plasma membrane. Some misfolded SLO-1 protein may be refolded in erg-28 sel-11 mutants and continues along the secretory pathway to the plasma membrane and the remaining misfolded SLO-1 is degraded via another SEL-11 independent pathway (maybe dependent on autophagy/lysosomes). The partial restoration of SLO-1 levels in the DNC and muscle in erg-28 sel-11 doubles in Fig 1 are consistent with this model.

Major comments

1. The fact that SLO-1 levels are not fully restored to wild type in sel-11 mutants (Fig 1) suggests that SLO-1 might be degraded via another pathway independent of sel-11. This data is consistent with a model where SLO-1 is misfolded in the absence of the chaperone erg-28 and degraded partly via a sel-11/ERAD mechanism and partly via another sel-11 independent pathway (perhaps via autophagy in the lysosome). Does inhibition of the proteasome and/or lysosome completely block the reduction in SLO-1 levels seen in erg-28 single mutants?

We do not think that ERG-28 is SLO-1 chaperone (We clarified this point in our responses to reviewer #1's major concern 1 and reviewer #2's concern 2). Rather, ERG-28 is important for SLO-1 exit from the ER. The absence of ERG-28 delays SLO-1 trafficking and causes SLO-1 channels to recognized as misfolded. [The role of ERG-28 is consistent with our prior observation that ERG-28 is a highly mobile protein with COPI binding sites (Oh et al, 2017). Deletion of the COPI binding site is sufficient to abolish ERG-28 function.] In our current and previous studies, we indeed did not observe the

complete recovery of SLO-1 levels by bortezomib (Oh et al, 2017) or *sel-11* mutations (current study). We believe that bortezomib cannot inhibit all of the proteasome activities; bortezomib blocks the growth and development of the treated animals and causes growth arrest or death in a high dose. We have tested the possible involvement of autophagy/lysosome, but we could not find it (this is addressed in Major comments 3). Our current hypothesis is that SEL-11 is a major protein responsible for SLO-1 degradation, and other ER-resident E3 ubiquitin ligases redundantly remove the rest of SLO-1 channels.

2. A major conclusion of this paper is that in the absence of the ER chaperone ERG-28, SLO-1 is normally folded yet still degraded via ERAD. Although the SLO-1(gf) experiments in Figure 2 suggest that the SLO-1(gf) channels are ultimately functional if ERAD is blocked in sel-11 mutants, these experiments do not definitively show that SLO-1 is normally folded in erg-28 mutants. How do the authors know that SLO-1 is not misfolded or improperly assembled in the absence of erg-28? The authors should tone down their conclusion that normally folded SLO-1 is degraded via ERAD and discuss the alternative that SLO-1 is simply misfolded in the absence of erg-28 and targeted for ERAD.

If we assume that, as the reviewer states, SLO-1 channels are misfolded in the absence of ERG-28, we cannot explain that misfolded SLO-1 channels become refolded in the *sel-11 erg-28* double mutant (In comparison to wild-type animals, 70-80% SLO-1 channels are lost in *erg-28* mutant, and over 60% SLO-1 channels are recovered in the *sel-11 erg-28* double mutants). Once proteins become misfolded and adopt a certain energetically stable conformation, they cannot be correctly refolded without the aid of a chaperone.

We performed additional experiments, which are described in our response to Reviewer #2's major concern 2. Misfolded SLO-1 channels will have a problem in exiting the ER and fail to incorporate into presynaptic terminals. Based on this idea, we compared the amount of overexpressed GFP-tagged SLO-1 that reaches to presynaptic terminals in wild-type and *erg-28* mutant animals but did not observe a significant difference, indicating that SLO-1 channels are not misfolded in *erg-28* mutant animals (line 223-230, S4 Fig).

We performed an additional aldicarb experiment to demonstrate that not only SLO-1(gf) channels, but also endogenous SLO-1 channels, are also degraded in the absence of ERG-28, and their function is restored by a *sel-11* mutation (this is also related to the reviewer's major comment 4), providing another piece of evidence that SLO-1 is normally folded in *erg-28* mutant. This data is presented in a new paragraph (lines: 214-222) and S3 Fig.

Our study is based on *C. elegans* genetic analysis. Given the low expression levels of SLO-1 channels in limited tissues (relying on the endogenous promoter), the biochemical study is difficult to perform. Based on the reviewer's comments, we toned down our conclusions and discussed alternative possibilities.
Abstract: Changed "normally folded" to "seemingly normally folded."

Discussion: Changed “GFP-tagged SLO-1 channels are normofolded, but misfolded” to “GFP-tagged SLO-1 channels do not appear to be misfolded in the *erg-28* mutant.” We also discuss an alternative mechanism (lines: 460-466) in Discussion.

3. The SLO-1(gf)::GFP levels in erg-28 sel-11(tm1743) mutants should be imaged and quantified in the dorsal cord or muscle to allow comparison to the effects of erg-28 sel-11(tm1743) on SLO-1::GFP in Figure 1. The partial restoration of SLO-1::GFP levels in sel-11 erg-28 mutants in Fig 1 and Fig 3D compared to the almost complete restoration of SLO-1(gf) function in Fig 2 suggests that much lower levels of SLO-1(gf) channels can fully restore function of SLO-1(gf) despite lower levels of expression. These results suggest that SLO-1(gf) function can be restored if some of the channels manage to properly fold/assemble in erg-28;sel-11 double mutants. These results also might suggest that misfolded BK channels that cannot be refolded in erg-28 sel-11 mutants ultimately get targeted for degradation via another sel-11 independent pathway.
First, we compared SLO-1::GFP levels in *slo-1(cim105)* and *slo-1(cim113gf)* animals and found no significant difference (this result is now added to Fig 3A). We showed that SLO-1(gf) levels are lower than wild-type SLO-1 levels in the *erg-28* mutant. Interestingly, we observed that the SLO-1 levels at the dorsal cord of *sel-11(tm1743) erg-28 slo-1(cim113gf)* animals (S5 Fig) are comparable to those of *sel-11(tm1743) erg-28 slo-1(cim105)* animals (Fig 1A) with ~200 fluorescent intensity units. This is discussed in a paragraph of Discussion (lines: 435-445). [Note that the images in the different figures are comparable because image acquisitions were performed with age-matched animals in the same settings.]

It is possible that SLO-1 is degraded in a *sel-11*-independent pathway, including the lysosome pathway. As explained above, we introduced an *atg-4.2* mutation to *erg-28 slo-1(cim105)* animals but were unable to find significant restoration of SLO-1 levels in *atg-4.2;erg-28* animals. This suggests that SLO-1 is not degraded by autophagy in *erg-28* mutants. Perhaps there is some partial redundancy within the ER-resident E3 ubiquitin ligases. It is possible that a single mutation in the other ER-resident E3 ubiquitin ligase genes is not sufficient to restore SLO-1 levels, but there may be some redundancy with different combinations of mutations. This is now discussed in Discussion (lines: 460-466).

4. If endogenous SLO-1 is degraded via ERAD in erg-28 mutants, then erg-28 mutants might be expected to have similar functional defects as slo-1 loss of function mutants. Do erg-28 mutants have functional defects (such as aldicarb or thrashing defects) that are similar to slo-1 loss of function mutants? And are these defects suppressed by sel-11?
Indeed, *erg-28* mutants exhibit reduction-of-function phenotypes; like *slo-1* mutant animals, *erg-28* mutant animals (without any other combined mutation) are hypersensitive to aldicarb (Oh et al, 2017) and exhibit acute tolerance to ethanol (Oh et al., 2019). They also exhibit hyperactive movement behavior (frequent backing) observed in *slo-1* loss-of-function mutants.

Minor comments

1. In Figure S4A and S4B, the cdc-48.2 genotypes under the graphs are incorrectly labeled cdc-48.1

We thank Reviewer #3 for pointing out this error. We modified Fig S7A and S7B to correctly label *cdc-48.2* (S4 Fig became S7 Fig).

2. In Figure 3A, it is not possible to conclude that SLO-1 is expressed at similar levels in cim105 and cim113gf because the GFP signal in these Nerve Ring images are saturated. Non-saturated images should be shown.

We agree with the reviewer. We compared the levels of SLO-1 at the dorsal cord and body wall muscles in both *slo-1(cim105)* and *slo-1(cim113gf)* and incorporated them into Fig 3A. In both the dorsal cords and body wall muscles, the levels of SLO-1 were similar.

3. Figure 3E is labeled slo-1(ky399gf) above the graph but referred to as slo-1(cim113gf) in the legend. Also, please clarify if the cim113gf point mutation is the same one found in the ky399gf allele.

We thank Reviewer #3 for pointing out this error. We have changed the incorrectly labeled *slo-1(ky399gf)* in Fig 3D and 3E to the appropriate name: *slo-1(cim113gf).*

4. Is SKN1A required for normal expression of proteasome genes? If SKN-1A is only involved in regulating proteasome gene expression after proteasome dysfunction, why does mutation of skn-1A suppress SLO-1 degradation? Does erg-28 mutation cause proteasome dysfunction?

A previous publication by Lehrbach et al. (2019) showed that *skn-1a* mutation indeed affects the basal level of the proteasome (Figure 5A and 5C in Cell 177, 7373-750). Therefore, there is a low, but present, level of SKN-1A that escapes degradation and is involved in the maintenance of the basal proteasome level. This is consistent with our observation that *skn-1a* mutation does affect SLO-1 degradation. We now referenced this paper in our discussion (line: 415)

To address whether an *erg-28* mutation causes proteasome dysfunction, we introduced *erg-28(gk697770)* mutation to the *rpt-3p::gfp* proteasome subunit reporter strain. We did not observe an increase in GFP fluorescence level, indicating that *erg-28* does not cause proteasome dysfunction. This result is now in a new paragraph (lines: 364-370) and S10 Fig.

5. Figure 6B suggests that activated SKN1A can induce further degradation of SLO-1 even in the absence of sel-11. It would be helpful if the authors discuss what other degradative pathways might be utilized.

We thank the reviewer for insightful comments. We now include a new paragraph in Discussion (lines: 460-466): “We note that there may be a minor *sel-11*-independent pathway for SLO-1 degradation, evidenced by the incomplete restoration of SLO-1 levels in *sel-11 erg-28* mutants. Since autophagy does not appear to contribute to SLO-1 degradation (S9 Fig), perhaps there is some partial redundancy among the ER-resident E3 ubiquitin ligases. It is possible that a single mutation in the other genes that encode the ER-resident E3 ubiquitin ligases is not sufficient to restore SLO-1 levels.

Still, a different combination of mutations with some redundancy may increase SLO-1 levels."

6. Does Bortezomib block the degradation of SLO-1 induced by activated SKN-1A shown in Figure 6B?

We treated *cimSi4;sel-11(tm1743) erg-28 slo-1(cim105)* animals with bortezomib. If bortezomib blocks the degradation of SLO-1 by the *skn-1a*-upregulated proteasomes, then these bortezomib-treated animals would exhibit higher levels of SLO-1 than non-treated. Bortezomib treatment did not alter SLO-1 levels at the dorsal cord, suggesting that bortezomib does not block the degradation of SLO-1 by *skn-1a*-upregulated proteasomes. We note that as reported by Lehrbach and Ruvkun (2016) a low dose of bortezomib treatment severely delays the growth and development of these animals due to the *sel-11* background, indicating that complete proteasome inhibition is not possible.

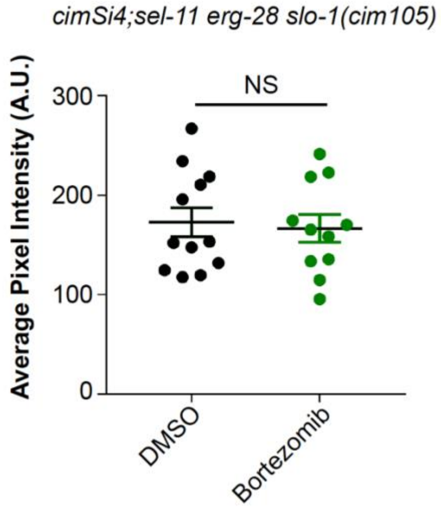

8. It would be interesting and surprising if SLO-1 is folded normally in erg-28 mutants but still degraded by ERAD. Although, the function of such a mechanism is not clear. The one example of a normally folded protein being degraded via ERAD cited in the discussion indicates that normally folded HMG-CoA reductase is degraded via ERAD in response to increased levels of cholesterol. The physiological function of degrading normally folded HMG-CoA reductase in response to increased cholesterol makes physiological sense because this is a negative feedback pathway where the cholesterol end product signals to degrade HMG CoA reductase, an enzyme that lies upstream in the biosynthetic pathway. In contrast, the physiological function of degrading normally folded SLO-1 in erg-28 mutants is not clear. The simplest explanation is that in the absence of the ER chaperone ERG-28, SLO-1 is misfolded or misassembled and thus degraded via ERAD. This alternative interpretation should be considered in the discussion.

We appreciate the reviewer's insightful comments. In response to the reviewer's comment, we added the following paragraph to Discussions. "The physiological

advantage of SLO-1 degradation in the ER remains unclear. ERG-28 normally facilitates anterograde SLO-1 trafficking from the ER, and its absence may increase SLO-1 retention time in the ER. The ER is a major storage compartment for calcium ions, and it releases calcium to the cytosol through ryanodine receptors in muscles and neurons. It is possible that the released calcium ions from the ER may bind to ER-retained SLO-1 channels and trigger a conformational change, which is readily recognized and targeted for degradation. This may also explain why an *erg-28* mutation causes lower levels of gain-of-function SLO-1 than those of normal SLO-1, and a combination of *erg-28* and *sel-11* results in comparable levels of both SLO-1 (Fig 1A and S5 Fig). Further investigation is required to understand how ERG-28 protects SLO-1 channels from degradation."

As the reviewer suggested, we also toned down our conclusion and provided alternative interpretations (major comments 2, 3).
